# 

**Published:** 1858-01

**Authors:** 


*Meeting of the Erie County Medical Society.*—The next meeting of the Erie Co. Med. Society, will take place at the rooms of the Buffalo Medical Association, on the second Tuesday in January. We hope that members from the country will bear this in mind, and that there will be a full attendance. The oration will be delivered by Dr. Newman. F.

---

*To Correspondents.*—Occasionally, through press of business, we omit to acknowledge the receipt of matter for publication from our contributors; and as it is frequently obliged to lie over for a number or two, the authors are uncertain whether they are accepted or rejected. We would here give notice that all communications which we do not receive, will be immediately returned with an explanation of our reasons; but matter which we intend to pnblish will be put on file and appear as soon as we have the space. F.

